# Giant Placental Chorangioma and Severe Ductal Arch Constriction: A Case Report With a Favorable Outcome

**DOI:** 10.7759/cureus.38209

**Published:** 2023-04-27

**Authors:** Claudio V Schenone, Alejandro Rodriguez, Jose Duncan, Thora Steffensen, J. Blaine John, Sarah Običan

**Affiliations:** 1 Obstetrics and Gynecology, University of South Florida, Tampa, USA; 2 Pathology, University of South Florida, Tampa, USA; 3 Pediatrics and Fetal Cardiology, St. Joseph's Children's Hospital, Tampa, USA

**Keywords:** polyhydramnios, fetus, ductal constriction, chorioangioma, giant chorangioma

## Abstract

Giant chorangiomas are uncommon yet frequently associated with adverse pregnancy outcomes. A 37-year-old female was referred due to findings of a placental mass during a second-trimester ultrasound. A fetal survey at 26 weeks revealed a 69×97×75 mm heterogenous placental tumor with two prominent feeding vessels. Her prenatal course was complicated by worsening polyhydramnios requiring amnioreduction, gestational diabetes, and transient severe ductal arch (DA) constriction. Placental pathology confirmed the diagnosis of giant chorioangioma following delivery at 36 weeks. To our knowledge, this represents the first case of DA constriction in the setting of a giant chorangioma.

## Introduction

Chorangiomas were first described by Clarke in 1798 [1]. These benign placental tumors comprise numerous small dense capillaries and loose fibrous connective tissue [2] and are mostly found incidentally after birth in 0.6% of gestations [3]. In contrast, giant chorangiomas, defined as >4-5 cm, are far rarer and commonly linked to fetal growth restriction (FGR), polyhydramnios, high output heart failure, fetal hydrops, and stillbirth [3-5], and high perinatal death rates that are, at least partly, driven by the paucity of evidence and lack of consensus for management [6]. We present a rare prenatally detected giant chorangioma associated with polyhydramnios and transient severe ductal arch (DA) constriction.

## Case presentation

**Figure 1:**
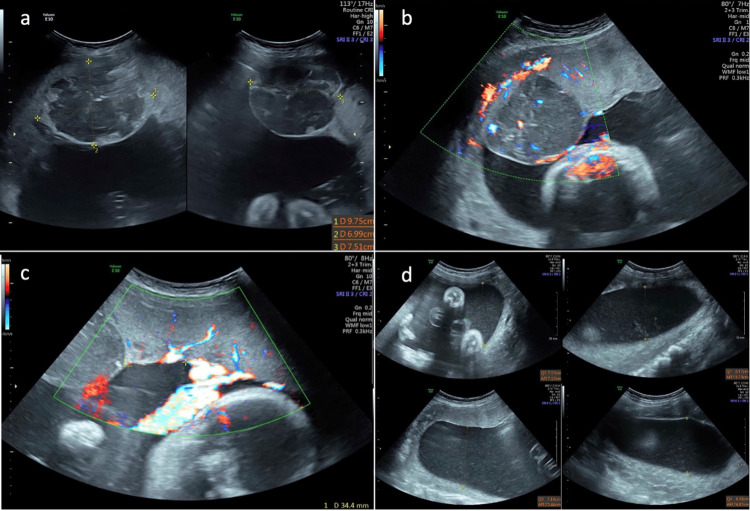
Ultrasound image at 26 weeks gestation showing a heterogeneous placental mass (a), tumor’s feeding vessels (b), distance from placental cord insert (c), and polyhydramnios (d).

A 31-year-old woman, gravida two, para one, was referred for findings suspicious of placental chorangioma. An ultrasound (US) at 26 weeks gestation showed a live fetus with an estimated fetal weight (EFW) at >99th percentile, polyhydramnios (amniotic fluid index {AFI} = 43.7 cm), and a well-defined, circumferential, heterogenous placental mass at the chorionic surface bulging into the amniotic cavity measuring 69×97×75 mm. On ultrasound, two prominent feeding vessels were located 34 mm away from the placental cord insert (Figures 1a-1d).

**Figure 2:**
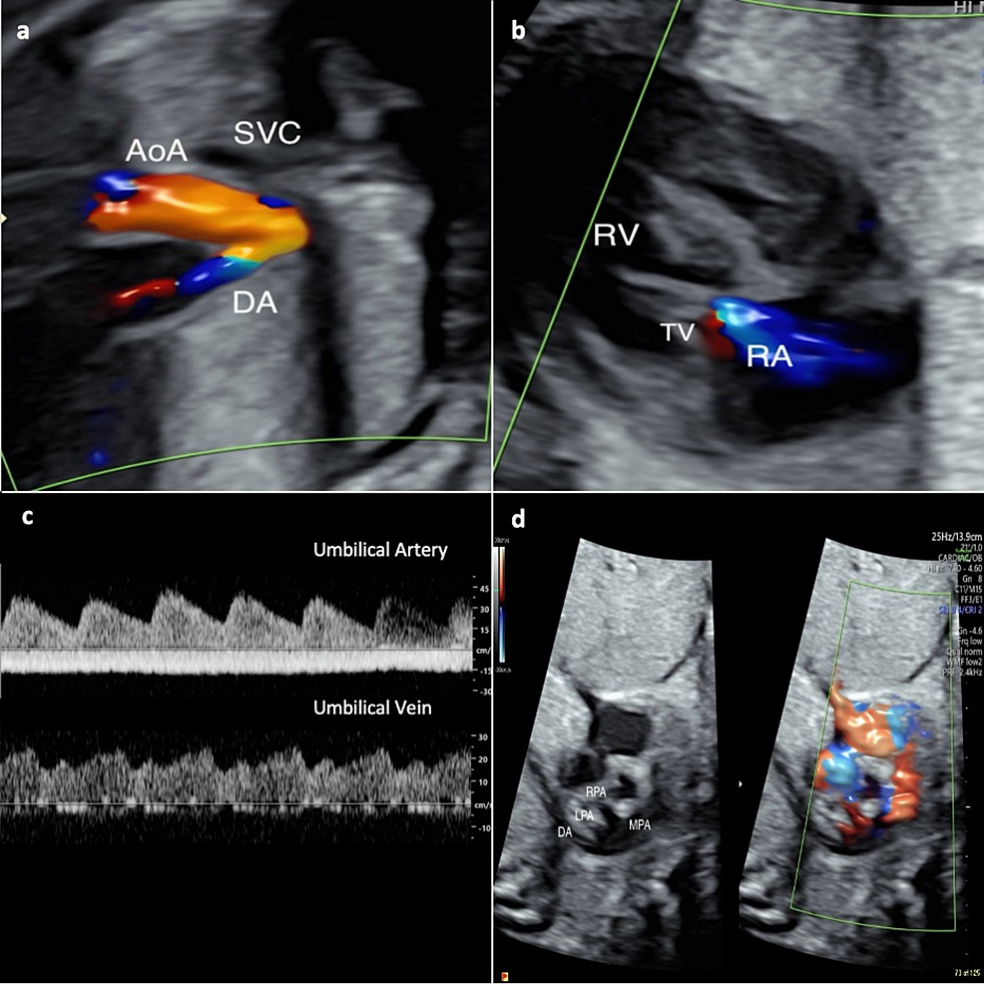
Fetal echocardiogram showing severe ductal arch constriction (a), moderate tricuspid regurgitation (b), normal umbilical vein and ductus venosus Dopplers (c), and follow-up examination showing widely patent ductus arteriosus (d).

The anatomic survey, including a fetal echocardiogram, and Doppler assessment of middle cerebral artery peak systolic velocity (MCA-PSV) and umbilical artery pulsatility index (UA-PI), was unremarkable. Given worsening abdominal discomfort, hospital admission for monitoring, antenatal steroids, and US-guided amnioreduction (AR) were recommended. Additionally, we discussed the availability of in-utero devascularization in the event of fetal hydrops and/or heart failure. A total of two liters of fluid was removed, and the post-procedure deepest vertical pocket (DVP) was 9 cm. The patient reported significant relief and was ultimately discharged home on hospitalization day 3. An amniotic fluid analysis reported a 46XX karyotype and normal microarray. Biophysical profile (BPP), fetal echocardiogram (FE), MCA-PSV, UA-PI, tumor size, and hydrops assessment were performed weekly thereafter, in addition to serial growth examinations. Her prenatal course was also complicated by gestational diabetes (GDM), which was well-controlled. A follow-up US at 29 weeks noted spontaneous resolution of previously diagnosed polyhydramnios. However, a repeat FE one week later (30 weeks) revealed new-onset severe DA constriction, moderate tricuspid valve regurgitation (TR), and right ventricle (RV) dysfunction, prompting re-admission for monitoring (Figures 2a-2d).

**Figure 3:**
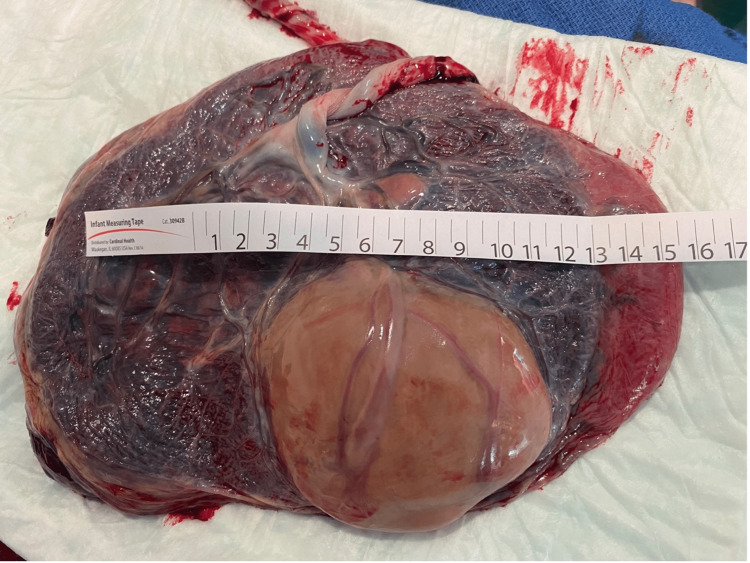
Macroscopic view of placental chorangioma.

**Figure 4:**
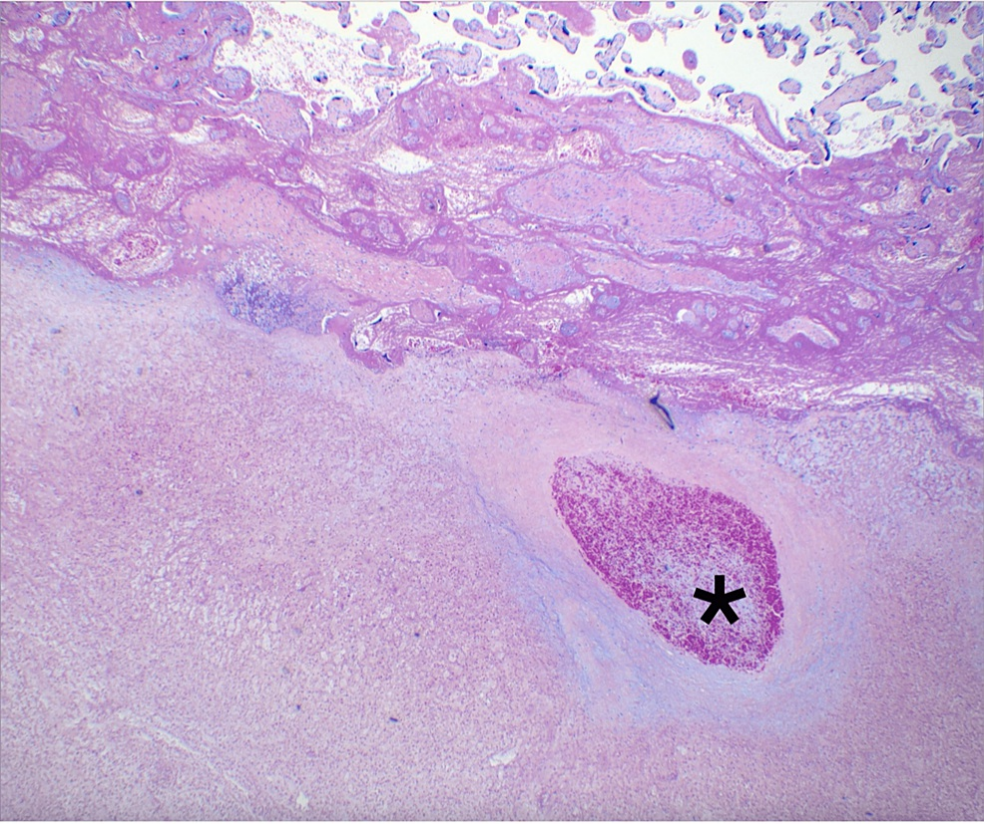
Microscopic view of chorangioma (lower half of image), with a mass of capillaries within stroma appearing as ghost cells, lacking cytologic detail. *An occlusive thrombus in organization within a large vessel that shows an infarcted, structureless wall (H&E stain, 40× magnification).

During her hospitalization, interval US at 32 weeks showed the absence of previously identified blood flow through the tumor’s feeding vessels, shrinking chorangioma (85×51×74 mm) and feeding vessels, and resolution of prior echocardiographic findings. Outpatient follow-up resumed, and labor induction was undertaken at 36 3/7 weeks after a thorough discussion with the patient and family members. A 2770 g baby girl was delivered vaginally with appearance, pulse, grimace, activity, and respiration (APGAR) scores of nine and nine at one and five minutes, respectively. An episode of neonatal hypoglycemia warranted a 24-hour admission to the neonatal intensive care unit. Otherwise, her course was uncomplicated, and she was discharged home on the second day of life. The placenta weighed 650 g (90-95th percentile). Gross examination revealed an 83×82×47 mm peripheral pink-gray-yellow variegated, rubbery, demarcated, subchorionic, infarcted mass, and histopathological examination confirmed the diagnosis of giant chorioangioma (Figures 3, 4).

The infant’s postnatal course remained uncomplicated at six months of life, meeting all developmental milestones. The patient's postpartum course was also unremarkable, including a normal 75 g 2 h oral glucose tolerance test (OGTT).

## Discussion

Chorangiomas arise from the chorionic mesenchyme due to aberrant vasculogenesis of mature stem villi. Prenatally identified tumors are most commonly detected in the late second trimester [7,8]. Our report is in line with previous authors reporting higher rates of female fetuses, GDM, and neonatal hypoglycemia in pregnancies complicated by chorangioma [2,3,9]. These tumors act as arteriovenous shunts that increase fetal cardiac output. Additionally, fetal blood sequestration within the tumor’s vascular array may cause microangiopathic hemolytic anemia [10]. Together, these mechanisms underlie the onset of common non-structural anomalies such as FGR, high-output heart failure, fetal anemia, fetal hydrops, and polyhydramnios [11]. The latter results from greater fluid transudation and increased urine production in response to hyperdynamic circulation [12,13]. In contrast, structural fetal anomalies in the setting of chorangiomas are much rarer. To our knowledge, this represents the first case of severe DA constriction in the setting of a prenatally detected giant chorangioma. Most cases of non-congenital heart defect and non-NSAID-related DA constriction are idiopathic or associated with a polyphenol-rich diet [14]. Our patient was counseled to avoid these substances during a prior FE and voiced compliance at diagnosis. It is unclear whether the presence of chorangioma and the onset of DA constriction in our case were coincidental. Previous studies have described spontaneous thrombosis and infarction in these tumors [15,16]. In our case, the co-occurrence of the absence of previously identified blood flow through the tumor’s feeding vessels (later confirmed on placental pathology), tumor shrinking, and DA constriction resolution point to this phenomenon as the underlying explanation, shedding light on a potential link between the two conditions. A similar occurrence has been reported in pregnancies with chorangioma-related complications other than DA constriction [8,17].

The scarcity of evidence has challenged the antenatal management of giant chorangiomas. The likelihood of perinatal death is positively correlated with tumor size and the presence of fetal hydrops [6]. Temporizing interventions include intrauterine transfusion and AR for cases complicated by fetal anemia and polyhydramnios, respectively. Many authors report fluid reaccumulation and recurrence of symptoms shortly after AR [12,18,19]. However, the amniotic fluid volume (AFV) in our case remained stable and ultimately normalized. In-utero tumor devascularization has gained popularity in recent years. However, it is unclear whether these procedures improve outcomes. Randomized controlled trials are lacking, as are studies comparing the different techniques. Furthermore, previous authors have brought up the issue of publication bias [6,20]. These factors highlight the need for multicenter data registries and additional evidence to further delineate the natural history of this condition, identify prognostic factors, develop staging systems, and compare outcomes based on different management strategies.

## Conclusions

Our case represents the first report of a giant placental chorangioma and severe DA constriction with favorable outcomes. Although the mechanistic link between these two conditions has yet to be discovered, this report highlights the importance of serial fetal echocardiograms in pregnancies complicated by these tumors to identify changes that may warrant closer monitoring to minimize complications. The rare nature of giant chorangiomas calls for further evidence to delineate their natural history and management strategies that help improve outcomes for these patients.
